# Identification of a novel functional deletion variant in the 5'-UTR of the DJ-1 gene

**DOI:** 10.1186/1471-2350-10-105

**Published:** 2009-10-13

**Authors:** Rowena J Keyser, Lize van der Merwe, Mauritz Venter, Craig Kinnear, Louise Warnich, Jonathan Carr, Soraya Bardien

**Affiliations:** 1 Division of Molecular Biology and Human Genetics, Faculty of Health Sciences, University of Stellenbosch, Cape Town, 8001, South Africa; 2 Biostatistics Unit, Medical Research Council, Cape Town, 8001, South Africa; 3 Department of Statistics, University of Western Cape, Cape Town, 8001, South Africa; 4 Department of Genetics, University of Stellenbosch, Stellenbosch, 7600, South Africa; 5 Division of Neurology, Faculty of Health Sciences, University of Stellenbosch, Cape Town, 8001, South Africa

## Abstract

**Background:**

DJ-1 forms part of the neuronal cellular defence mechanism against oxidative insults, due to its ability to undergo self-oxidation. Oxidative stress has been implicated in the pathogenesis of central nervous system damage in different neurodegenerative disorders including Alzheimer's disease and Parkinson's disease (PD). Various mutations in the *DJ-1 *(*PARK7*) gene have been shown to cause the autosomal recessive form of PD. In the present study South African PD patients were screened for mutations in *DJ-1 *and we aimed to investigate the functional significance of a novel 16 bp deletion variant identified in one patient.

**Methods:**

The possible effect of the deletion on promoter activity was investigated using a Dual-Luciferase Reporter assay. The *DJ-1 *5'-UTR region containing the sequence flanking the 16 bp deletion was cloned into a pGL4.10-Basic luciferase-reporter vector and transfected into HEK293 and BE(2)-M17 neuroblastoma cells. Promoter activity under hydrogen peroxide-induced oxidative stress conditions was also investigated. Computational (*in silico*) *cis*-regulatory analysis of *DJ-1 *promoter sequence was performed using the transcription factor-binding site database, TRANSFAC via the PATCH™ and rVISTA platforms.

**Results:**

A novel 16 bp deletion variant (g.-6_+10del) was identified in *DJ-1 *which spans the transcription start site and is situated 93 bp 3' from a Sp1 site. The deletion caused a reduction in luciferase activity of approximately 47% in HEK293 cells and 60% in BE(2)-M17 cells compared to the wild-type (*P *< 0.0001), indicating the importance of the 16 bp sequence in transcription regulation. The activity of both constructs was up-regulated during oxidative stress. Bioinformatic analysis revealed putative binding sites for three transcription factors AhR, ARNT, HIF-1 within the 16 bp sequence. The frequency of the g.-6_+10del variant was determined to be 0.7% in South African PD patients (2 heterozygotes in 148 individuals).

**Conclusion:**

This is the first report of a functional *DJ-1 *promoter variant, which has the potential to influence transcript stability or translation efficiency. Further work is necessary to determine the extent to which the g.-6_+10del variant affects the normal function of the *DJ-1 *promoter and whether this variant confers a risk for PD.

## Background

The *DJ-1 *gene (*PARK7*; OMIM 602533) was first described about a decade ago [1] and encodes a 189 amino acid protein which belongs to the DJ-1/Thi/PfpI protein super family [2,3]. It is ubiquitously expressed in a variety of mammalian tissues including the brain, and was initially described in association with oncogenesis and male rat infertility [1,4,5]. Later it was shown to be associated with autosomal recessive early-onset Parkinson's disease (PD) [3,6]. A few PD-causing mutations have been identified including exon deletions, truncations, homozygous and heterozygous point mutations, which predominantly result in loss of function [3,7]. The expression of DJ-1 in the central nervous system (CNS) is not restricted to specific anatomical or functional systems and it is located in neuronal and glial cells within the substantia nigra [8,9].

*DJ-1 *is proposed to play a role in protecting neurons from oxidative stress and protecting against mitochondrial damage [10,11]. Oxidative stress can be defined as an imbalance between reactive oxygen species (ROS) production and the antioxidant capacity of a cell. Mitochondrial dysfunction leading to increased ROS can cause damage to various cellular components such as unsaturated lipids, proteins, and nucleic acids, and this has been implicated in various neurodegenerative disorders including Alzheimer's disease, amyotrophic lateral sclerosis and PD [12]. Recent studies aimed at elucidating the function of DJ-1, have found that it is able to undergo self oxidation in order to eliminate hydrogen peroxide (H_2_O_2_) and, in so doing, acts as a scavenger of ROS [11,13]. An increase in susceptibility to oxidative stress of mammalian cells in cases where DJ-1 was knocked down has been reported [10,11]; cells were more susceptible to H_2_O_2_-induced cell death due to DJ-1 knockdown and over-expression of wild-type DJ-1 rescued the cells [14]. It has been reported that DJ-1 is oxidatively damaged in the brains of PD patients [15,16]. Studies showed that under oxidative stress conditions, DJ-1 undergoes a shift in its isoelectric point which leads to the accumulation of acidic isoforms in PD frontal cortex tissue compared to age-matched controls [15]. Up-regulation of DJ-1 as well as the intracellular redistribution of DJ-1 to the mitochondria under oxidative stress conditions has been reported [10,17]. The mitochondria have a central role in free radical generation and it has been proposed that DJ-1 in the mitochondria may have a role in preventing mitochondrial injury or decreasing mitochondrial ROS production. Redistribution of DJ-1 to the mitochondria might be the neuronal cellular defence mechanism against oxidative insults [9,10,18].

*DJ-1 *is highly conserved across diverse species [19] and there have been limited reports of sequence variants. An 18 bp insertion/deletion polymorphism (g.168_185del) in *DJ-1*'s promoter region has been reported to not confer a risk for PD [20,21]. However, a homozygous duplication of this 18 bp sequence (g.168_185dup), as well as a homozygous E163K mutation, have been identified in an Italian family with a Parkinsonism-Dementia-Amyotrophic Lateral Sclerosis phenotype [22]. The duplication and the E163K variants were not found in 1,400 and 500 control chromosomes, respectively. It has been reported that the E163K mutation compromises the ability of DJ-1 to protect against oxidative stress induced by H_2_O_2 _[23]. It is not known whether the phenotype in this family was due to the g.168_185dup mutation or the E163K mutation, or both.

In the present study, we investigated the functional significance of a novel 16 bp deletion which we identified in the promoter region of *DJ-1 *in a PD patient by means of luciferase functional expression studies. Unlike the 18 bp insertion/deletion variant, the 16 bp deletion occurs in a transcriptionally important region of the gene as it spans the transcription start site.

## Methods

### Study participants

The study protocol was approved by the Committee for Human Research at the University of Stellenbosch, South Africa (Protocol number 2002/C059). In the initial part of the study 30 unrelated South African PD patients were recruited with informed written consent from the Movement Disorders clinic at Tygerberg Hospital in South Africa. The patients were examined by a movement disorder specialist (JC) and all met the UK Parkinson's Disease Society Brain Bank Research criteria for diagnosis of PD [24]. Inclusion criteria for recruitment of patients were early onset and/or positive family history of PD. The average age at onset (AAO) of the study group was 43 years and ranged from 17 years to 77 years. The percentage of males in our study group was 66%. Once the 16 bp deletion variant was detected, the frequency of the variant was determined in a total of 148 unrelated South African PD patients (average AAO = 52 years). Blood samples for 62 controls representing the mixed ancestry ethnic group were recruited from unrelated blood donors at the South African Western Province Blood Transfusion Service blood collection clinics. The controls had been 'de-identified' and had not been clinically assessed for signs of PD.

### Genetic analysis

The single-strand conformational polymorphism analysis method was used to screen the 30 PD patients for genetic variations in all 7 exons and the 5' UTR of *DJ-1*. Samples exhibiting altered mobilities on mildly-denaturing polyacrylamide gels (containing 5% glycerol and 15% urea) were sequenced in order to characterize the sequence variation. Direct sequencing was performed using the BigDye Terminator Sequence Ready Reaction kit version 3.1 (Applied Biosystems, Foster City, USA) and analysis was conducted on a 3130 xl Genetic Analyser (Applied Biosystems).

### Construction of luciferase vectors

Functional analysis of a novel 16 bp deletion identified in the 5'-UTR was performed using a Dual-Luciferase Reporter Assay System . In this assay, the activities of the firefly (*Photinus pyralis*; the experimental reporter gene) and the *Renilla *(*Renilla reniformis *or sea pansy; the internal control gene) luciferases are measured sequentially from a single sample. The firefly luciferase gene is present on the promoterless pGL4.10 [*luc2*] vector and the *Renilla *luciferase gene is on the phRL-SV40 vector. The pGL4 luciferase reporter vector was used in this study because it has been codon optimized for more efficient expression in mammalian cells. The reporter gene and the vector backbone have been engineered to reduce the number of consensus transcription factor binding sites, which leads to reduced background luminescence and the risk of anomalous transcription. In this experiment the pGL4.10 [*luc2*] and phRL-SV40 vectors are used to co-transfect mammalian cells and due to their distinct evolutionary origins the activity of two luciferases can be distinguished since they have different enzyme structures and substrate requirements.

The promoter region of *DJ-1 *containing sequence spanning the 16 bp deletion was PCR amplified from genomic DNA from the individual harbouring the deletion variant using primers containing restriction sites for cloning. Primers were designed to PCR amplify a fragment from position 1 to 2119 bp (GenBank accession number AB045294). It was decided to include the entire 5'UTR sequence in the luciferase assay to ensure that all sites important for transcription initiation and regulation, especially under hydrogen peroxide-induced oxidative stress conditions, were present. A similar sized construct had previously been used successfully to assess promoter function of *DJ-1 *in a study done by Taira *et al*., 2001 [19].

Two constructs were generated; wild-type [pDJ-1(wt)Luc] and a 16 bp deletion variant [pDJ-1(del)Luc]. Primer sequences used were: forward 5'-ATC GTA TCG CTC GAG GGA TCC TTC TAA GCT CAT TC-3' and reverse 5'-CAG AGC TCT TTT GGA AGC AAG CTT CGATACGAT-3'. PCR reactions were performed in a 2720 Thermal Cycler (Applied Biosystems). The 50 μl reactions contained 200 ng template DNA (wild-type or deletion), 20 pmoles of each primer, 75 μM dNTPs (Promega), 1.5 mM MgCl_2_, 1× NH4 buffer (Bioline), 5% DMSO and 0.5 units of BIOTAQ DNA polymerase (Bioline). The PCR cycling parameters entailed an initial denaturation step at 95°C for 7 min, followed by 30 cycles of denaturation at 95°C for 30 sec, annealing at 50°C for 30 sec and extension at 72°C for 2 min, and a final extension step at 72°C for 10 min. The 2,151-kb sized PCR products (either wild-type sequence or 16 bp deletion sequence), as well as pGL4.10 [*luc2*] were digested with *XhoI *and *HindIII *(Fermentas) and purified (Wizard SV Gel and PCR Clean-up system, Promega). Vector arms were dephosphorylated (CIP, Promega) and ligated overnight to the digested PCR fragments. The pDJ-1(wt)Luc and pDJ-1(del)Luc constructs were subcloned into E. coli DH5α cells and single colonies were miniprepped (GeneJET plasmid miniprep kit, Promega). Insertion of the *DJ-1 *promoter sequence in-frame with the firefly luciferase gene in the pGL4.10 [*luc2*] vector was verified by direct sequencing using the BigDye Terminator Sequence Ready Reaction kit version 3.1 (Applied Biosystems) and analyzed on a 3130 xl Genetic Analyzer (Applied Biosystems).

### PCR-restriction fragment length polymorphism (RFLP) analysis

PCR-restriction fragment length polymorphism (RFLP) analysis was used to screen 148 PD patients and 62 control samples for the presence of the 16 bp deletion variant. The control samples were of South African mixed ancestry descent which can be defined as an admixture of indigenous African populations and immigrants from mainly Western Europe, Madagascar, Malaysia and India. The following PCR primers were designed which span the 16 bp deletion: forward 5'-ACC CAG GGC TGT CCA GCT A-3' and reverse 5'-GTC CAG CAC AGG GAC ACC-3' and produced a PCR product of 321 bp and 305 bp for the wild type and deletion alleles, respectively. A total of 8 μl of the PCR product was digested overnight at 37°C with 5 units of *KpnI *(Promega) in a final volume of 20 μl. Thereafter, the digested products were electrophoresed on 12% polyacrylamide gels and the bands visualized by silver staining. Following the *KpnI *digest, the samples could be genotyped since the wild type allele produced two fragments of 129 bp and 192 bp whereas the 16 bp deletion allele produced two fragments of 129 bp and 176 bp.

### Cell culture

Human embryonic kidney cells (HEK293) (Highveld Biological, Pty, Ltd, South Africa) and human dopaminergic neuroblastoma BE(2)-M17 cells (American Type Culture Collection, USA) were cultured separately under sterile conditions in Dulbecco-modified Eagle medium (LONZA BioWhittaker^®^) supplemented with 10% fetal calf serum, penicillin (100 units/ml), and streptomycin (100 μg/ml) in 5% CO_2 _humidified atmosphere at 37°C.

### Plasmid transfection and luciferase assay

Cultured HEK293 and M17 cells were plated 24 hrs prior to transfection into 6-well culture dishes at 80-90% confluence and maintained at an atmosphere of 5% CO_2 _at 37°C. For transfections, 1 μg of DNA was added with 3 μl Fugene (Roche Biochemicals) and serum-free media to a total volume of 100 μl (DNA/Fugene/media complex). The complex was then added to the cells in the culture dishes in a drop-wise manner. The pDJ-1(wt)Luc and pDJ-1(del)Luc constructs were co-transfected with phRL-SV40, an internal control vector containing the *Renilla *luciferase gene (Promega), in a ratio of 50:1 (pGL4 versus phRL-SV40), in order to control for transfection efficiency. Twenty four hours after transfection, the cells were exposed to H_2_O_2 _(0-75 μM), in order to produce ROS formation. Forty eight hours after transfection, the cells were gently rinsed with phosphate buffered saline and harvested with Passive Lysis Buffer (Promega). The Dual-luciferase^® ^Reporter Assay System (Promega) was used to measure luciferase activity. Twenty microliters of cell lysate were added to 100 μl of luciferase assay reagent II (Promega) and the firefly luminescence was read using a Modulus 96 Luminometer. Next, 100 μl of Stop&Glo^® ^reagent (Promega) was added to the lysates and renilla luminescence was read. Luminescence values of firefly were normalized with renilla for each construct within an experiment. The luciferase assay was repeated in four independent experiments for each cell type and the luminescence readings were read in triplicate.

### Clonogenic survival assay

A clonogenic survival assay was conducted in order to determine the appropriate concentration of H_2_O_2 _to use in the luciferase assay. The cultured HEK293 and M17 cells were plated in appropriate dilutions (1000-2000 cells/well) into 6-well plates, and incubated for 48 hrs. The cells were then exposed to varying concentrations of H_2_O_2 _(0, 25, 50 and 75 μM) and incubated for approximately one week. Thereafter, the cells were fixed and stained with 0.2% crystal violet (Merck) overnight for visual colony count. The IC_50 _values (concentration of H_2_O_2 _required to inhibit colony formation by 50%) were determined for both cell lines.

### Bioinformatics

Computational (*in silico*) *cis*-regulatory analysis of *DJ-1 *promoter sequence was performed using the transcription factor-binding site database, TRANSFAC via the PATCH™ platform . For PATCH™ analysis, the primary parameter settings were set as: Minimum Length of Sites at 4 and Lower Score Boundary at 85%. The multi-species conserved sequence analyses were performed using the rVISTA (regulatory VISTA) platform  at default cut-off criteria: ≥ 70% identity over a contiguous paired sequence length ≥ 100 bp. rVISTA combines searching the major transcription factor binding site database TRANSFAC with a comparative sequence analysis. *DJ-1 *promoter sequences (2 kb upstream and 5'-UTR) from human (Ensembl Gene ID: ENSG00000116288; *H. sapiens*), chimpanzee (ENSPTRG00000000102, *P. troglodytes*), orangutan (ENSPPYG00000001925, *P. pygmaeus*), macaque (ENSMMUG00000019671, *M. mulatta*), tree shrew (ENSTBEG00000009474, *T. belangeri*), horse (ENSECAG00000017251, *E. caballus*), dog (ENSCAFG00000019674, *C. familiaris*), cow (ENSBTAG00000020518, *B. taurus*), mouse (ENSMUSG00000028964, *M. musculus*) and rat (ENSRNOG00000018289, *R. norvegicus*) were retrieved from the Ensembl Genome Browser (archive version at ).

### Statistical analysis

The open-source programming environment R (freely available from ) was used for the statistical analysis. A linear mixed-effects model was created to analyse the data. All the relative light units (RLU) values were first log-transformed towards symmetry, and then modelled as a function of the factors including cell line (HEK293 or M17), Type (WT or deletion) and H_2_O_2 _concentration (0, 25, 50 or 75 μM) as fixed effects. We also included interactions between Type and cell line, as well as between Type and H_2_O_2 _concentration, both of which were independently significant after adjusting for all the other effects. As the individual experiments yielded RLU values that were dissimilar, each experiment were included in the model as a random effect. All the results reported here come from the same model, and were therefore adjusted for all the other factors in the model, as well as the random effect of individual experiments.

## Results

During mutation screening of South African PD patients a novel 16 bp deletion in the promoter region of *DJ-1 *was identified. The frequency of this deletion (g.-6_+10del) was estimated to be 0.7% (95% confidence interval 0.2% to 2.4%), based on 2 heterozygotes in 148 South African PD patients screened. Both patients were sporadic individuals of South African mixed ancestry. One of the patients presented with PD at age 38 years. He had displayed early onset of prominent autonomic dysfunction and hallucinations (with psychosis) and had died at the age of 47 years. The other patient had an age at onset of 56 years and also has a history of psychotic episodes. Furthermore, the g.-6_+10del variant was observed in a homozygous state in one of 62 mixed ancestry control individuals screened (1.6%). This individual was aged 34 years and had not been clinically assessed for signs of PD.

**Figure 1:**
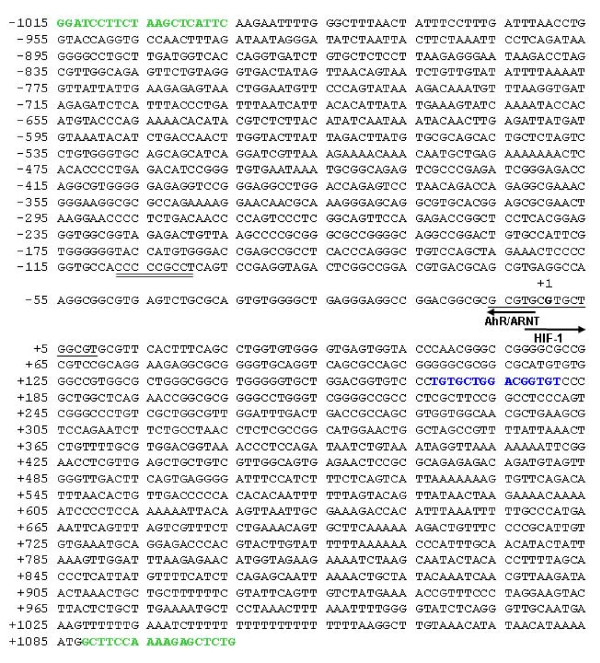
**Nucleotide sequence of the promoter region of the human *DJ-1 *gene (GenBank Accession number **AB045294**) showing the positions of significant sites**. The +1 indicates the transcription start site. The position of the 16 bp deletion sequence is indicated by a single underline and the Sp1 site by a double underline. The position of the 18 bp insertion/deletion polymorphism (g.168_185del) is indicated in blue font and the positions of the primers used to generate the luciferase constructs are shown in bold green font. The positions of putative *cis*-motifs (AhR/ARNT and HIF-1) present in the 16 bp deletion sequence are indicated by arrows.

The 16 bp deletion spans the transcription start site of *DJ-1 *which had previously been identified by Taira *et al. *[19] using the method of 'CAP site hunting'. This variant is situated 93 bp downstream of a Sp1 site, which has been shown to be necessary for *DJ-1 *transcription [19], and 157 bp upstream from the known g.168_185del polymorphism (Figure 1). We hypothesized that this deletion would disrupt transcription of DJ-1 and this hypothesis was tested using a luciferase reporter assay. DJ-1 has previously been shown to be expressed in the HEK293 cell line [19] and these cells were therefore used in the initial experiments.

To determine the *in vitro *effect of the deletion variant on *DJ-1 *promoter activity, two different constructs (wild-type and deletion variant) were generated. The promoter region of *DJ-1 *containing the sequence spanning the 16 bp deletion, as well as transcriptionally important sites such as the transcription start site and Sp1 site, was linked to the firefly luciferase gene and the luciferase activity was measured in RLU. Both constructs contained the 18 bp of the polymorphic variant (g.168_185del). The results obtained are therefore likely to be due only to the presence or absence of the 16 bp sequence.

The linear mixed-effects model for log(RLU) values, showed a significant interaction between construct type and H_2_0_2 _concentration (p = 0.0010) and also between construct type and cell line (p = 0.0119). All three factors: cell line, construct type and H_2_0_2 _concentration, had highly significant effects (p < 0.0001) on log(RLU) after adjusting for the interactions and experiments. All the P-values presented in the present study come from this model and they are therefore adjusted for the differences between the individual experiments as well as all the other factors in the model [the cell line (HEK293 or M17), construct type (WT or deletion) and H_2_O_2 _concentration (0, 25, 50 or 75 μM) as well as the interactions between Type and cell line, and between Type and H_2_O_2 _concentration]. We compared the geometric means (the mean of the logs, which is then anti-logged) of the observed RLU values obtained for each combination of the M17 cells, the construct type and the H_2_O_2 _concentration to the predicted means generated by the model (results not shown). These results indicated that the model fitted the data well and we therefore have confidence in our results.

**Figure 2:**
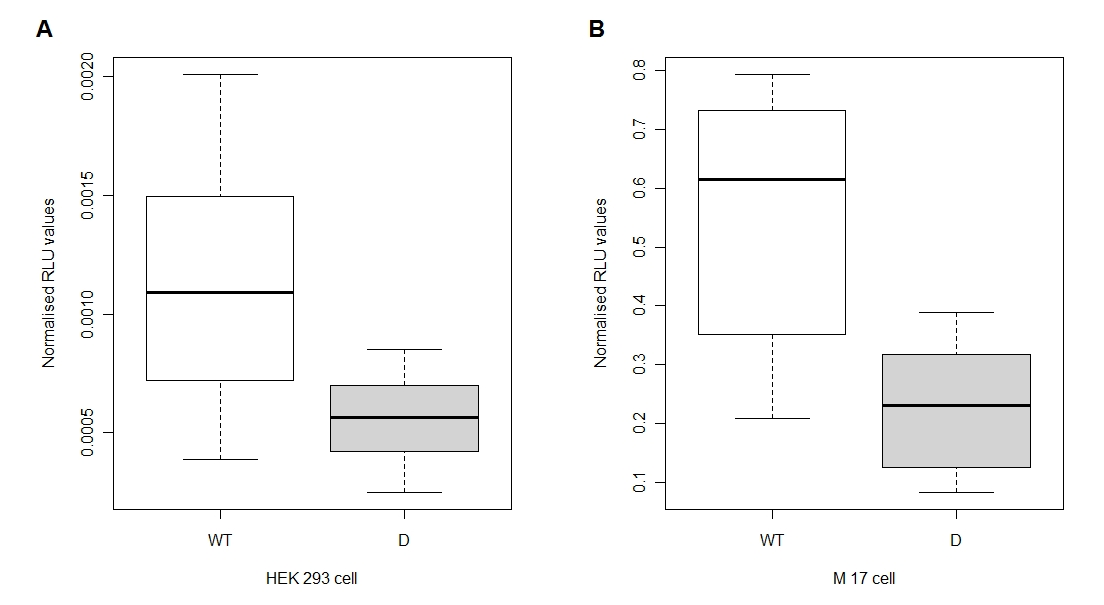
**The *DJ-1 *16 bp deletion variant exhibited significantly reduced transcription levels compared to the wild-type (P < 0.0001) in two different cell lines**. Box plots of dual-luciferase assays of the wild-type and deletion variant using (A) HEK293 cells and (B) neuroblastoma M17 cells. They include median (horizontal line within box), inter-quartile interval i.e. 25^th ^to 75^th ^percentile (box) and the range of variation (whiskers). The 2 kb promoter fragments (containing either wild-type sequence or the deletion) were linked to the luciferase gene and the constructs were transfected into two mammalian cell lines. For each experiment, firefly luciferase activity was divided by renilla activity to normalize for transfection efficiency. Data are representative of four transfection experiments (triplicate points obtained over four independent experiments). WT: wild-type; D: deletion variant; RLU: relative light units.

The wild-type construct produced consistently higher luciferase activity compared to the activity of a promoterless vector, which indicated that the insert contained a functional promoter (results not shown). Figure 2 shows the distribution of the normalized RLU values obtained for the wild-type and deletion variant in both cell lines. There was a significant difference between the wild-type and the deletion variant in the HEK293 cell line, after adjusting for experiments and all other factors. The deletion caused a reduction in luciferase activity of approximately 47% compared to the wild-type, indicating that the 16 bp sequence is probably important for the transcriptional regulation of *DJ-1 *(Figure 2A). Conducting the assay in M17 neuroblastoma cells (Figure 2B) produced similar results with an even larger reduction of 60% in luciferase activity observed for the deletion compared to the wild-type. From the box plots of the data, it was noted that for both cell lines, approximately 75% of the RLU values of the deletion variant were less than the lower 25% quartile of the RLU values for the wild-type (Figure 2), which illustrates the significant difference in the RLU values between the two constructs.

### H_2_O_2 _treatment of the M17 neuroblastoma cells

The effect of the deletion variant on *DJ-1 *promoter activity under H_2_O_2_-induced oxidative stress conditions was investigated using M17 neuroblastoma cells. DJ-1 is a scavenger of ROS and is proposed to play a role in protecting neurons from oxidative stress. A clonogenic survival assay was first performed to assess the concentration of H_2_O_2 _to use for these experiments. The IC_50 _values (concentration of H_2_O_2 _required to inhibit colony formation by 50%) were shown to be 50 μM for both cell lines (results not shown). The H_2_O_2 _concentration of 75 μM had the highest cytotoxic effect on both cell lines.

**Figure 3:**
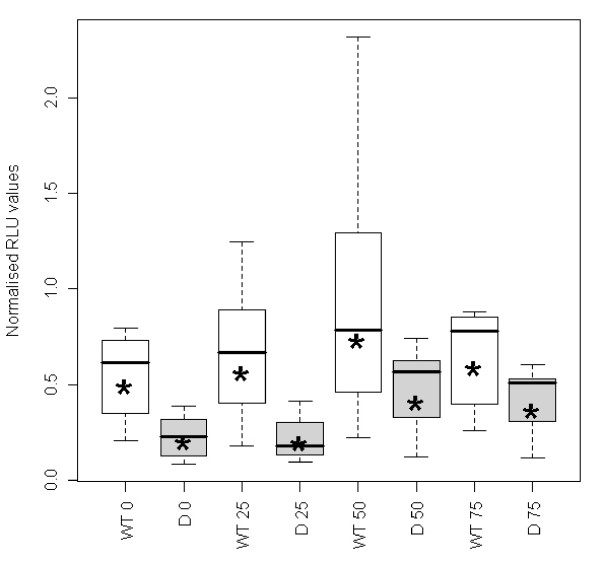
**H_2_O_2 _dose-dependent up-regulation of DJ-1 promoter activity**. Box plots of the dual-luciferase assay of the 16 bp *DJ-1 *deletion variant using the neuroblastoma M17 cell line and exposure to different concentrations of H_2_O_2 _(0, 25, 50 and 75 μM). *: geometric means; WT: wild-type; D: deletion variant; 0, 25, 50, 75: H_2_O_2 _concentrations.

Under H_2_O_2_-induced oxidative stress conditions, the deletion variant again exhibited significantly reduced promoter activity compared to the wild-type for the untreated as well as at all three concentrations of H_2_O_2 _(Figure 3). The promoter activity of both the wild-type and deletion was moderately upregulated during these oxidative stress conditions with increasing concentrations of H_2_O_2_. However, this trend was not observed at the highest H_2_O_2 _concentration of 75 μM, possibly because of increased cell death at this dosage. Although both the wild-type and deletion exhibited increased promoter activity with increasing concentrations of H_2_O_2_, the promoter activity for the deletion variant was always lower than that of the wild-type. In a post-hoc analysis with H_2_O_2 _as dichotomous [treated (all concentrations > 0) versus untreated] we found a highly significant difference (p < 0.0001) in promoter activity between untreated cells and H_2_O_2 _treated cells (after adjusting for all other factors in model, namely the construct type, the cell line and the H_2_O_2 _concentrations (0, 25, 50 or 75 μM) and the interactions between type and each of the other two factors).

### Computational cis-regulatory analysis of DJ-1

Interestingly, the 16 bp deletion sequence contains three perfect 5'-GCGT-3' and one imperfect repeat (5'-GCTG-3'). The functionality of this particular motif is currently not known. Analysis of the 16 bp deletion sequence (using the TRANSFAC database via PATCH and rVISTA) revealed the over-representation of three putative *cis*-motifs that correlate to the transcription factors Aryl hydrocarbon receptor (AhR; TRANSFAC Acc. Nr. T00018), Ahr nuclear translocator (ARNT; T01797) and Hypoxia induced factor 1 (HIF-1; T01609) within the 16 bp sequence (Figure 1). The core binding sequence for AhR and ARNT is 5'-CACGC-3' and for HIF-1 is 5'-RCGTG-3'. These transcriptional regulators control a variety of developmental and physiological events including metabolism of toxins and responses to hypoxia [25,26], and share regulatory cross-talk with many other transcription factors. When the 16 bp sequence is deleted a transcription factor site is created. *In silico *promoter analysis revealed that the sequence in the (-) strand (GCGCGTTC) of the 16 bp deleted region conforms to a novel *cis*-motif that putatively correlated to *cis*/*trans *interaction with the E2F-class of transcription factors.

**Figure 4:**
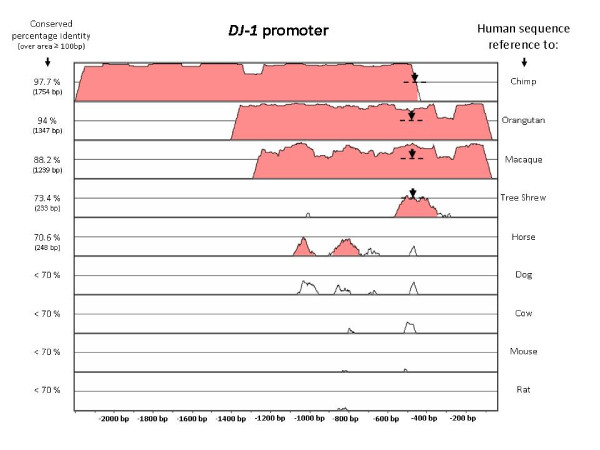
**Comparative multi-species analysis using rVISTA of the *DJ-1 *promoter region of approximately 2000 bp + 5'-UTR**. Conserved percentage identity is indicated with cut-off criteria: ≥ 70% identity over ≥ 100 bp are indicated in orange and the white regions represent either no alignment or ≤ 70% sequence identity over 100 bp contiguous sequence length. The 16 bp deletion sequence is represented by an arrow on a dashed line and is found in a region sharing highly conserved identity between species.

Multi-species comparative analysis using rVISTA, [27], showed regions of ≥ 70% conserved identity (over contiguous sequence length: 100 bp) of the human *DJ-1 *promoter region to chimpanzee (1754 bp), orangutan (1347 bp), macaque (1239 bp), tree shrew (233 bp) and horse (248 bp). All the other species investigated including rat and mouse showed ≤ 70% conservation (Figure 4).

## Discussion

In the present study we report a novel 16 bp deletion (g.-6_+10del) in the promoter region of *DJ-1*. The deletion was shown to span the transcription start site, according to Taira *et al. *2001 [19] and is situated 93 bp downstream of a Sp1 transcription regulatory sequence which is essential for *DJ-1 *promoter activity. In addition, the g.-6_+10del variant is located 157 bp upstream from a known 18 bp duplication mutation (g.168_185dup). The duplication mutation has previously been reported to co-segregate, together with a homozygous E163K mutation, with early-onset Parkinsonism-Dementia-Amyotrophic Lateral Sclerosis in an Italian family [22]. Due to its location within the DJ-1 promoter, we hypothesised that the 16 bp deletion variant might influence the level of DJ-1 expression, the transcript stability or the translation efficiency.

Functional expression studies using the dual-luciferase reporter system found that the g.-6_+10del variant significantly reduced *DJ-1 *promoter activity in both HEK293 and neuroblastoma M17 cells, with the M17 cells exhibiting a higher reduction in activity. This indicates the possible importance of the 16 bp sequence in transcriptional regulation of *DJ-1*. Recent studies aiming to elucidate the function of DJ-1, have identified it as a scavenger of ROS due to its ability to undergo self oxidation in order to eliminate H_2_O_2 _[11]. It was therefore decided to assess the effect of the deletion variant on *DJ-1 *promoter activity under H_2_O_2_-induced oxidative stress conditions using the neuroblastoma M17 cell line. In the present study it was shown that cells placed under oxidative stress conditions showed a dose-dependent moderate up-regulation in both wild-type and deletion variant's promoter activities with the deletion variant retaining its lower RLU values compared to the wild-type.

Furthermore, bioinformatic analysis identified binding sites for the transcription factors; Aryl hydrocarbon receptor (AhR), Ahr nuclear translocator (ARNT) and Hypoxia induced factor 1 (HIF-1) within the 16 bp deletion sequence. AhR and ARNT are known to dimerize to form an active transcription factor complex that binds defined DNA sequences, the xenobiotic-responsive element (XRE), with high affinity causing an increase in transcription of AhR-regulated genes [26,28,29]. The AhR/ARNT complex up-regulates cytochrome P450 enzymes that play diverse roles in metabolism of endogenous substances, environmental chemicals and various drugs [29]. AhR functions as the prime transcription factor and ARNT as a DNA binding partner. Transcriptional up-regulation during hypoxia is mediated principally by HIF-1 (a dimer of HIF-1α and ARNT). The anticancer property of curcumin has been shown to be due to inactivation of HIF-1 by degradation of ARNT via oxidation and ubiquitination processes [30]. It was also shown that curcumin induces proteasomal degradations of both AhR and ARNT and this is mediated by oxidative stress [31]. It is possible that the AhR/ARNT or HIF-1 complexes may interact with DJ-1's promoter thereby regulating its transcription and therefore indirectly influencing the transcription of various genes associated with oxidative stress, apoptosis and neurotoxicity that are regulated by DJ-1 [32].

Further analysis found that when the 16 bp sequence is deleted, a binding site for the E2F-class of transcription factors is created. E2F has been shown to induce transcription of pro-apoptotic proteins and repression of E2F-responsive genes is required for neuronal survival [33]. Recent studies found that the pRb/E2F cell-cycle pathway is activated in dopaminergic neurons in PD patients, as well as in a PD mouse model [34].

DJ-1 is highly conserved at both the nucleotide and amino acid level, possibly due its important role in protection against oxidative stress. Mouse and human DJ-1 is 83% and 90% identical at the DNA and protein level, respectively [19]. Similarly, the conservation between porcine and human DJ-1 is 96% at the protein level [35]. There have been no reports however on the level of conservation of DJ-1's 5'-UTR sequence. We showed through multi-species comparative analysis that the promoter region of *DJ-1 *is highly conserved, as expected, between man and other primates as well as to a few other species. More importantly, the 16 bp deletion sequence is present in a highly conserved promoter region (Figure 4) indicating the possible significance of this sequence in higher order mammals and thus an evolutionarily retained functionality.

It would be interesting to determine the prevalence of the g.-6_+10del variant in other populations and to establish whether it plays a role in susceptibility to neurodegenerative diseases such as PD. Further studies are necessary to determine whether endogenous levels of DJ-1 are affected by the 16 bp deletion and whether an alternative transcriptional start site is present. In addition, introducing point mutations into the predicted transcription factor binding sites within the 16 bp sequence to determine the effect on DJ-1's transcription and the cell's response to oxidative stress would be of interest.

## Conclusion

We report a novel sequence variant in the highly conserved DJ-1 gene. Functional expression studies found that this variant significantly reduced *DJ-1 *promoter activity in two separate mammalian cell lines, which indicates the possible importance of the 16 bp sequence in transcriptional regulation of *DJ-1*. In addition, the activity of three transcription factors with recognition sites within the deletion sequence might be influenced by the g.-6_+10del variant.

## Competing interests

The authors declare that they have no competing interests.

## Authors' contributions

RJK performed the laboratory work and drafted the manuscript. LvdM performed the statistical analysis. MV conducted the bioinformatic analysis. LW and JC critically reviewed the manuscript. CK assisted with laboratory work and critically reviewed the manuscript. SB participated in the conception and design, and helped to draft the manuscript. All authors read and approved the final manuscript.

## Pre-publication history

The pre-publication history for this paper can be accessed here:


